# The roles of sclerostin and irisin on bone and muscle of orchiectomized rats

**DOI:** 10.1186/s12891-022-05982-7

**Published:** 2022-12-02

**Authors:** Bing-na Zhou, Qian Zhang, Xiao-yun Lin, Jing Hu, Di-chen Zhao, Yan Jiang, Xiao-ping Xing, Mei Li

**Affiliations:** Department of Endocrinology, National Health Commission Key Laboratory of Endocrinology, Peking Union Medical College Hospital, Chinese Academy of Medical Sciences and Peking Union Medical College, Shuaifuyuan No. 1, Dongcheng District, Beijing, 100730 China

**Keywords:** Sclerostin, Irisin, Osteoporosis, Sarcopenia, Orchiectomy

## Abstract

**Background:**

The reduction in androgen level gives rise to a decrease in bone mineral density (BMD) and muscle strength, but the exact mechanisms are unclear. We investigated the roles of novel cytokines of sclerostin and irisin on bone and muscle of orchiectomized rats.

**Methods:**

Twenty 3-month-old male rats were randomized to receive sham or orchiectomy (ORX) operation. Rats were euthanized after 8 weeks of surgery, and serum levels of sclerostin and irisin were measured by enzyme-linked immunosorbent assay at baseline and execution. Grip strength was measured by a grip strength tester at baseline and before execution. BMD and bone microarchitecture were measured by microcomputed tomography. The samples of bone and muscle were harvested at execution. Bone biomechanics were measured by three-point bending tests and vertebral body indentation tests. Bone and muscle histological features were analyzed by hematoxylin and eosin stain, Von Kossa’s stain and tartrate resistant acid phosphatase stain. Simple linear regression analyses were used to analyze the relationships between serum levels of sclerostin, irisin and grip strength and BMD of ORX rats.

**Results:**

Serum sclerostin level increased from 279 ± 44 pg/mL to 586 ± 57 pg/mL since baseline to 8 weeks after ORX (*P* = 0.002), which was significantly higher than that in sham rats (406 ± 20 pg/mL at execution) (*P* = 0.012). Serum irisin level decreased from 4.12 ± 0.20 ng/mL to 3.55 ± 0.29 ng/mL since baseline to 8 weeks of ORX (*P* = 0.048), which was significantly lower than sham rats (4.84 ± 0.37 pg/mL at execution) (*P* = 0.013). Trabecular BMD, parameters of bone microarchitecture, bone strength, grip strength and the myofibers size of soleus muscles were significantly lower in ORX rats than in sham group. Grip strength was positively correlated with femoral trabecular BMD (*r* = 0.713, *P* < 0.001) and bone volume/total volume (*r* = 0.712, *P* < 0.001) in all rats. The serum sclerostin level was negatively correlated to femoral trabecular BMD (*r* = -0.508, *P* = 0.022) and grip strength (*r* = -0.492, *P* = 0.028). Serum irisin level was positively correlated with femoral trabecular BMD (*r* = 0.597, *P* = 0.005), but no obvious correlation was found between irisin level and muscle strength in all rats.

**Conclusions:**

Reduced BMD, impaired bone microarchitecture, weak strength of bone and muscle, and thin myofibers were induced by androgen deficiency of ORX rats. Serum sclerostin and irisin levels were significantly changed after ORX, which might be closely correlated with the occurrence of osteoporosis and sarcopenia in ORX rats.

**Supplementary Information:**

The online version contains supplementary material available at 10.1186/s12891-022-05982-7.

## Background

With the ageing process in men, osteoporosis and sarcopenia have become more and more common diseases. Osteoporosis is a skeletal disease characterized by low bone mineral density (BMD) and bone microarchitecture disruption, resulting in loss of bone strength and increased fracture risk [1]. Sarcopenia is a progressive and generalized skeletal muscle disorder with loss of muscle mass and function [2]. Various situations such as ageing or pathological states, as well as environmental factors (nutrition, physical activity/mechanical stress) may influence muscle and bone simultaneously. As a matter of fact, muscle/bone relationships include different levels of crosstalk [3–5]. In systemic humoral pathways, androgen deficiency is important, which can lead to decreases in volume and strength of muscle and bone [6, 7], and they can be reversed by androgen replacement therapy [8, 9]. Androgen can influence bone and muscle volume and strength by binding directly to androgen receptors, or indirectly to estrogen receptors via testosterone aromatization to estrogen [8, 10, 11]. However, the exact molecular mechanism of osteoporosis co suffering with sarcopenia is still unknown in ageing men.

Muscle and bone are tightly coupled, not only in their anatomical proximity and mechanical interaction but also in terms of paracrine and endocrine signals [3, 5, 12]. Recently, muscle and bone are acknowledged to function in an endocrine manner to be involved in the muscle–bone crosstalk, and several kinds of osteokines and myokines attract increasing attention in the mechanisms of osteoporosis and sarcopenia [13]. Sclerostin is an osteokine, which inhibits bone formation and plays a role in skeletal muscle regeneration, insulin resistance, and glucose metabolism through Wnt signaling [14, 15]. Irisin, which is a myokine mainly secreted by skeletal muscle after physical exercise, has been proved to exert anabolic effects in several tissues such as bone, muscle, brain and so on, emphasizing the significance of physical exercise in delaying the onset of age-related diseases [16, 17]. For skeletal muscle, irisin exerts its autocrine action by enhancing the expression of the precursors from whose cleavage it derives [18]. For bone cells, irisin can increase osteoblast proliferation and differentiation through the Wnt/β-catenin pathway, and inhibit osteoclast differentiation by suppressing the receptor activator of nuclear factor-kappa B ligand (RANKL)/ nuclear factor of activated T cells 1 (NFATc1) pathway [19, 20]. These findings suggest that sclerostin and irisin may participate the crosstalk of muscle and bone [3, 17]. However, the roles of these two new cytokines in osteoporosis and sarcopenia are still unclear in men.

To identify a possible role of sclerostin and irisin in osteoporosis and sarcopenia of men, we evaluated the serum sclerostin and irisin levels in rats with orchiectomy (ORX) or sham operation, and analyzed the correlations of the serum sclerostin, irisin levels and BMD and grip strength in rats.

## Materials and methods

### Animals and experimental design

Twenty 3-month-old male Sprague–Dawley (SD) rats (weighing about 400-450 g) were acclimated for two weeks in standard cages in an Association for Assessment and Accreditation of Laboratory Animal Care (AAALAC) accredited animal facility in Peking Union Medical College Hospital (PUMCH). They were allowed free access to water and food. The animals were kept and handled under specific pathogen-free (SPF) conditions with controlled ambient temperature (21 ± 2 °C) and lighting (12 h light–12 h dark) conditions.

All rats were randomly divided into sham (*n* = 10) and ORX (*n* = 10) groups using a random number table. The sample size for each group was determined referring to some previous studies [7, 21]. SD rats underwent bilateral orchidectomy or sham surgical procedure after they were in narcotism by injecting 3% pentobarbital sodium into cavum abdominis (30 mg/kg). At baseline, 4 and 8 weeks after surgery, body weight of the rats were measured. Eight weeks after sham or ORX operation, the rats were euthanized by the intravenous injection of pentobarbital sodium (200 mg/kg). Blood, bone and muscle tissue samples were collected at execution.

All animal research procedures were approved by the Animal Welfare & Ethics Committee of PUMCH (No. for the application: XHDW-2022–039). All sections of the present study complied with the “ARRIVE guidelines” for reporting in vivo experiments in animal research. Completed “ARRIVE guidelines” checklist was included in Supplementary 1.

### Measurement of sclerostin and irisin levels

The blood samples of rats were collected, centrifuged, aliquoted, and immediately frozen at -80 °C. Serum sclerostin levels at baseline and at execution were determined by enzyme-linked immunosorbent assay (ELISA) kit for rat sclerostin (Cat. No. SEC864Ra, USCN Life Science, Wuhan, China) according to the manufacturer’s instructions. The intra- and inter-assay coefficients of variation (CVs) were less than 10 and 12% for sclerostin, respectively. Serum irisin levels at baseline and at execution were measured by an ELISA kit following the manufacturer's instructions (Cat. No. CSB-EL008770RA, Cusabio, Wuhan, China), with the intra- and inter-assay CVs less than 8 and 10%, respectively. All samples were analyzed in duplicate. Standard curves were created and the serum levels of sclerostin and irisin for each rat were calculated by Curve Fit.

### Measurement of serum testosterone and bone biochemical parameters levels

Serum testosterone level was measured with an automated Roche electrochemiluminescence system (Roche Diagnostics, Switzerland). Serum levels of calcium (Ca), phosphorus (P), total alkaline phosphatase (ALP, a marker of bone formation), and creatinine (Cr) were measured using an automatic biochemistry analyzer (Cobas Integra 400 plus, Roche kit) [22]. A commercial ELISA kit (Cat. No. CSB-E12776r, Cusabio, Wuhan, China) was used to detect the levels of C-telopeptide of type I collagen (CTX-I, a bone resorption marker) according to the manufacturer’s instructions, with intra-assay and inter-assay CVs both less than 15%.

### Evaluation of bone microarchitecture and BMD

Bone microarchitecture and BMD were evaluated using micro-computed tomography (μCT) as previously described [23]. After euthanasia with excess intravenous injection of pentobarbital sodium, the left femurs of the rats were dissected and fixed with 4% paraformaldehyde (PFA) at 4 °C for 24 h. Trabecular architecture of the distal femur were measured by μCT (Inveon MM CT, Siemens, Erlangen, Germany). Scans were operated with an X-ray tube voltage of 60 kV, a current of 400 μA, an exposure time of 800 ms and a voxel size of 20 μm. The region of interest (ROI) for trabecular bone was drawn in the area of 1.5 mm below the growth plate of the distal epiphysis, and extending 100 slices to proximal end. Cortical bone was analyzed in a 1000-μm-long volume situated in the middle of the diaphysis. Every 5 sections were outlined, and the intermediate sections were interpolated with the contouring algorithm to create a volume of interest. Segmentation values used for analysis were determined using the Inveon Research Workplace software (Siemens). A three-dimensional (3-D) analysis was performed to determine bone volume / tissue volume (BV/TV), bone surface area / bone volume (BS/BV), trabecular number (Tb.N), trabecular thickness (Tb.Th), trabecular separation (Tb.Sp) and trabecular pattern factor (Tb.Pf). A two-dimensional (2-D) analysis was performed to determine trabecular BMD. The mean cortical thickness (Ct.Th) was determined by distance measurements at 4 different points on the cortical slice of the left femurs.

### Measurement of bone biomechanical markers

The right femurs and L5 vertebral body excised immediately after euthanasia were frozen at -20 °C in plastic bags. The right femurs were thawed at room temperature for the three-point bending test as previously described [24]. The three-point bending tests were performed with the anterior surface of the femur resting on the bottom supports (span length: 20 mm) and the central load point moving at a rate of 1 mm/min until the bone fractured, with force and displacement data collected. The yield load, maximum load, breaking load, and stiffness were calculated from the load–displacement curve. Yield load was a force when the femur began to deform during compression, maximum load was the maximum force during the test, breaking load was a force when the femur totally broke during compression, and stiffness was the slope of the load–displacement curve.

For vertebral indentation tests, the end-plates and posterior and transverse elements of the L5 vertebral body were removed using a rongeur, thereby creating samples with a uniform 5 mm height and planoparallel ends [21]. Samples were then loaded in indentation by an indenter with 1 mm diameter at a rate of 1 mm/min. Force and displacement data were used to determine yield load, maximum load and stiffness.

Three-point bending tests and vertebral body indentation tests were completed using a Material Testing System (ElectroForce® 3200 Series III test instruments, TA Instruments, Waters Corporation, New Castle, DE).

### Evaluation of bone histological features

Bone histological features were analyzed by hematoxylin and eosin (H & E) stain, Von Kossa’s stain and tartrate resistant acid phosphatase (TRAP) stain. The left femurs and L3-4 vertebral body were used to analyze bone histology. Part of the bone samples was decalcified using ethylenediaminetetraacetic acid (EDTA, Servicebio, Wuhan, China). Decalcification was carried out for 8 weeks, and the decalcified bones were dehydrated and processed to form paraffin blocks. Paraffin sections of 4.0 µm thick were stained with H & E. The 4-µm-thick sections were also stained with TRAP to label osteoclasts. H & E and TRAP sections were photographed under a microscope (NIKON ECLIPSE E100, Nikon, Japan) with a CCD camera (NIKON DS-U3, Nikon, Japan). The other part of bone samples was processed as undecalcified specimens, which were dehydrated and embedded in polymer methyl methacrylate (MMA, M813511, Macklin) and sectioned at 10.0 µm thickness using a microtome (HistoCore AUTOCUT, Leica, Wetzlar, Germany). The sections were then stained using Von Kossa’s method for structural histomorphometry.

### Measurement of grip strength

At the baseline and eight weeks after sham or ORX operation, the grip strength of the rat limbs was measured using a grip strength tester (YLS-13A, ShangHai Biowill Co., Ltd., China, Fig. 3a). Rats grasped the grasping board by the four limbs, and then they were pulled continuously at a rate of approximately 1 cm/s. The instrument will automatically record the maximum grip strength of the rats. This test was performed 5 times and the results represented the average of each rat.

### Analysis of muscle histological features

After euthanasia, soleus muscles were removed from rats after 8 weeks of sham or ORX operation, which were fixed with 4% PFA for at least 24 h at 4 °C. The muscles were dehydrated and then embedded in paraffin. Sections of 4.0 µm thick were obtained, deparaffinized in xylene followed by rehydration in the sequence of 100, 100, and 75% ethanol for 5 min, and stained with H & E. H & E sections were photographed under a microscope with a CCD camera, and cross-sectional areas of at least 100 myofibers were quantified using ImageJ in a blinded manner.

### Statistical analysis

Data were analyzed using Student’s t test when two groups were compared. A paired-samples Student’s t test was used to longitudinally compare the differences in continuous variables between baseline and after sham or ORX operation. A simple regression analysis was conducted using Spearman’s rank correlation tests to analyze the associations of serum sclerostin, irisin levels and femoral trabecular BMD and grip strength. All quantitative data were presented as mean ± SEM.

Statistical analysis was performed using SPSS software, version 26.0 for Windows (SPSS Corp, Chicago, IL) and GraphPad PRISM 8.00 software. All tests were two-tailed, and *P* value of 0.05 was considered as statistical significance.

## Results

### Change of serum testosterone, sclerostin and irisin levels after ORX or sham surgery

The serum testosterone of experimental rats was measured at 8 weeks of ORX or sham operation. The serum testosterone level was 0.75 ± 0.04 ng/mL in ORX rats, which was significantly lower than that in sham rats of 7.98 ± 1.43 ng/mL (*P* < 0.001), indicating that rat models with androgen deficiency were successfully generated (Table 1).

**Table 1:** Bone biochemical, microarchitecture and biomechanical properties of rats after 8 weeks of ORX or sham operation

	ORX (*n* = 10)	Sham (*n* = 10)	*P* value
T (ng/mL)	0.75 ± 0.04	7.98 ± 1.43	**< 0.001**
Ca (mmol/L)	2.39 ± 0.02	2.41 ± 0.02	0.458
Cr (umol/L)	25.2 ± 0.95	25.2 ± 0.74	0.974
P (mmol/L)	2.06 ± 0.08	2.13 ± 0.05	0.503
ALP (U/L)	150.4 ± 9.5	122.6 ± 5.2	**0.020**
CTX-I (pg/mL)	47.52 ± 2.00	41.32 ± 1.06	**0.014**
Bone microarchitecture and BMD			
BV/TV (%)	26.1 ± 2.3	39.0 ± 2.5	**0.001**
Tb.N (1/mm)	13.66 ± 1.18	20.18 ± 1.26	**0.001**
BS/BV (1/mm)	104.69 ± 0.37	103.52 ± 0.22	**0.016**
Tb.Th (mm)	0.01917 ± 0.00007	0.01928 ± 0.00003	0.167
Tb.Sp (mm)	0.0628 ± 0.0117	0.0319 ± 0.0031	**0.021**
Tb.Pf (1/mm)	22.38 ± 1.01	18.87 ± 0.63	**0.009**
Ct.Th (mm)	0.77 ± 0.01	0.79 ± 0.02	0.150
Trabecular BMD (mg/cm^2^)	379.85 ± 12.44	527.43 ± 16.88	**< 0.001**
Whole femur three-point bending tests			
Yield load (N)	110.42 ± 4.86	125.06 ± 4.27	**0.036**
Maximum load (N)	152.67 ± 7.45	170.71 ± 3.02	**0.045**
Breaking load (N)	143.50 ± 6.49	162.39 ± 2.70	**0.020**
Stiffness (N/m)	89.82 ± 3.83	126.25 ± 12.61	**0.019**
Vertebral indentation tests			
Yield load (N)	31.16 ± 4.21	44.91 ± 4.22	**0.033**
Maximum load (N)	45.22 ± 4.99	60.96 ± 4.85	**0.036**
Stiffness (N/m)	64.20 ± 3.24	86.75 ± 8.80	**0.027**

Data expressed as mean ± standard error of the mean (SEM)

Bold values indicate that there was a signification difference between the two groups

*Abbreviations*: *ORX* Orchiectomy, *T* Serum testosterone, *Ca* Serum calcium, *Cr* Creatinine, *P* Serum phosphate, *ALP* Alkaline phosphatase, *CTX-I* C-telopeptide of type I collagen, *BV/TV* Bone volume per total volume, *Tb.N* Trabecular number, *BS/BV* Bone surface area per bone volume, *Tb.Th* Trabecular thickness, *Tb.Sp* Trabecular separation, *Tb.Pf* Trabecular pattern factor, *Ct.Th* Cortical thickness, *BMD* Bone mineral density

**Fig. 1:**
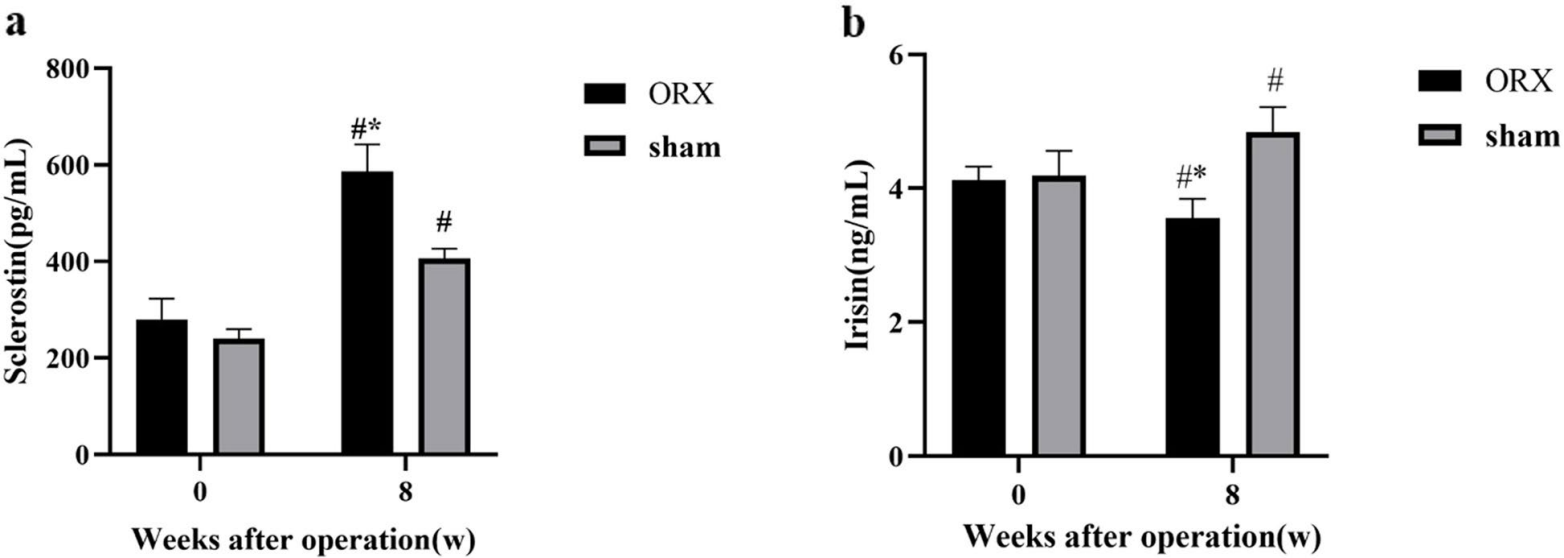
Change of serum sclerostin and irisin levels after ORX or sham operation. **a** Change of serum sclerostin levels in ORX and sham groups. **b** Change of serum irisin levels in the ORX and sham groups. *: *P* < 0.05 ORX vs the sham group after 8 weeks of operation. #: *P* < 0.05 vs baseline. Data expressed as mean ± standard error of the mean (SEM). Abbreviations: ORX: orchiectomy

At baseline, serum sclerostin concentration was 279 ± 44 pg/mL and 240 ± 20 pg/mL in ORX and sham group (*P* > 0.05). After 8 weeks of ORX or sham operation, serum sclerostin level significantly increased to 586 ± 57 pg/mL in ORX group, which were significantly higher than 406 ± 20 pg/mL of sham group (all *P* < 0.05, Fig. 1a).

Serum level of irisin was 4.12 ± 0.20 ng/mL and 4.19 ± 0.37 ng/mL in ORX and sham group at baseline (*P* > 0.05). After 8 weeks of operation, serum irisin level was 3.55 ± 0.29 ng/mL, which was significantly lower than 4.84 ± 0.37 ng/mL of sham group (*P* < 0.05, Fig. 1b).

### Changes of bone properties after ORX

The serum levels of ALP and CTX-I were significantly higher in ORX group than those in sham group after 8 weeks of operation, which indicated that the bone turnover was accelerated after ORX (Table 1). Serum levels of Ca, P and Cr were similar between the two groups (Table 1).

Impaired bone microarchitecture and reduced BMD were found in femur of ORX rats compared with the sham rats after 8 weeks of operation (Table 1). Trabecular BMD in the ORX rats was 379.85 ± 12.44 mg/cm^2^, which was markedly lower than that in sham group (527.43 ± 16.88 mg/cm^2^, *P* < 0.05) (Table 1). The 3-D analysis indicated that the BV/TV and the Tb.N of the ORX rats was decreased by 33.08% and 32.31% (both *P* = 0.001), whereas BS/BV was increased by 1.13% and Tb.Sp increased by 96.86% (all *P* < 0.05), Tb.Pf increased by 18.60% (*P* = 0.009) compared with the sham rats after 8 weeks of ORX or sham operation. No significant differences were noted in the Tb.Th and Ct.Th between the ORX and sham groups.

The strength and stiffness of femur were significantly lower in ORX rats than those in sham group after 8 weeks of ORX (*P* < 0.05, Table 1). The elastic load, maximum load, and stiffness at the L5 vertebral body were also significantly decreased in ORX rats than those in sham group after 8 weeks of ORX or sham operation (all *P* < 0.05, Table 1).

**Fig. 2:**
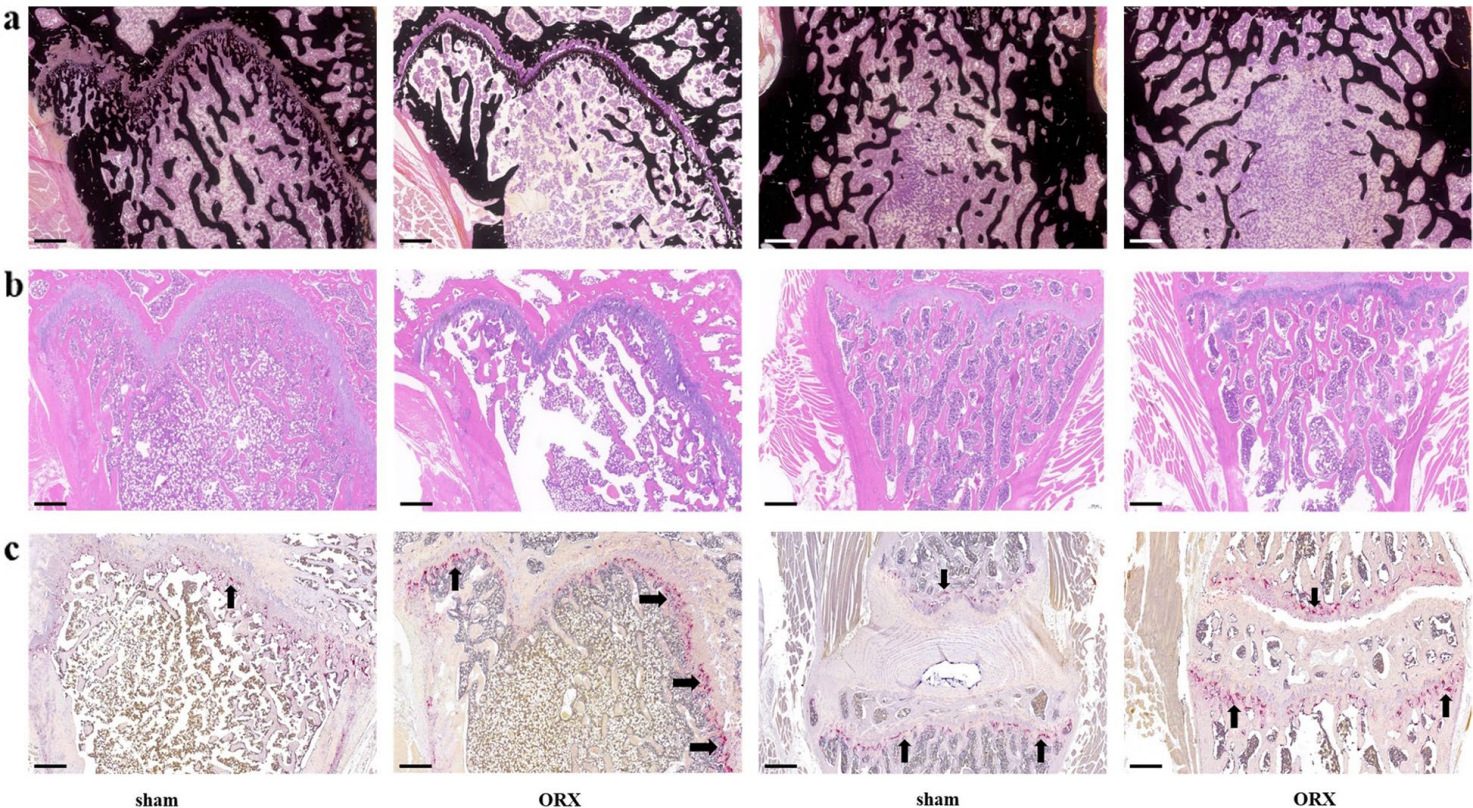
Bone histological characteristics of ORX and sham rats. **a** Von Kossa’s staining of the femur and the L3-4 in the ORX and sham group. **b** H&E staining of the femur and the L3-4 in the ORX and sham group. **c** TRAP staining of the femur and the L3-4 in the ORX and sham group. Scale bars indicate 500 µm. Arrows indicate the osteoblasts. Abbreviations: H&E: hematoxylin and eosin; TRAP: tartrate resistant acid phosphatase; ORX: orchiectomy

The Von Kossa’s (Fig. 2a) and H&E (Fig. 2b) staining images displayed the femoral and vertebral histology of the rats. The lumbar vertebral and femoral micrographs of the ORX group showed sparse loss of the trabecular interconnectivity and thinning of the trabecular, resulting in widened intertrabecular spaces over time. Histochemical localization of TRAP activity was evaluated in femur and L3-4 vertebral body (Fig. 2c), which indicated that the number of osteoclasts was significantly increased in ORX rats than sham rats.

### Changes of muscle properties after ORX

**Fig. 3:**
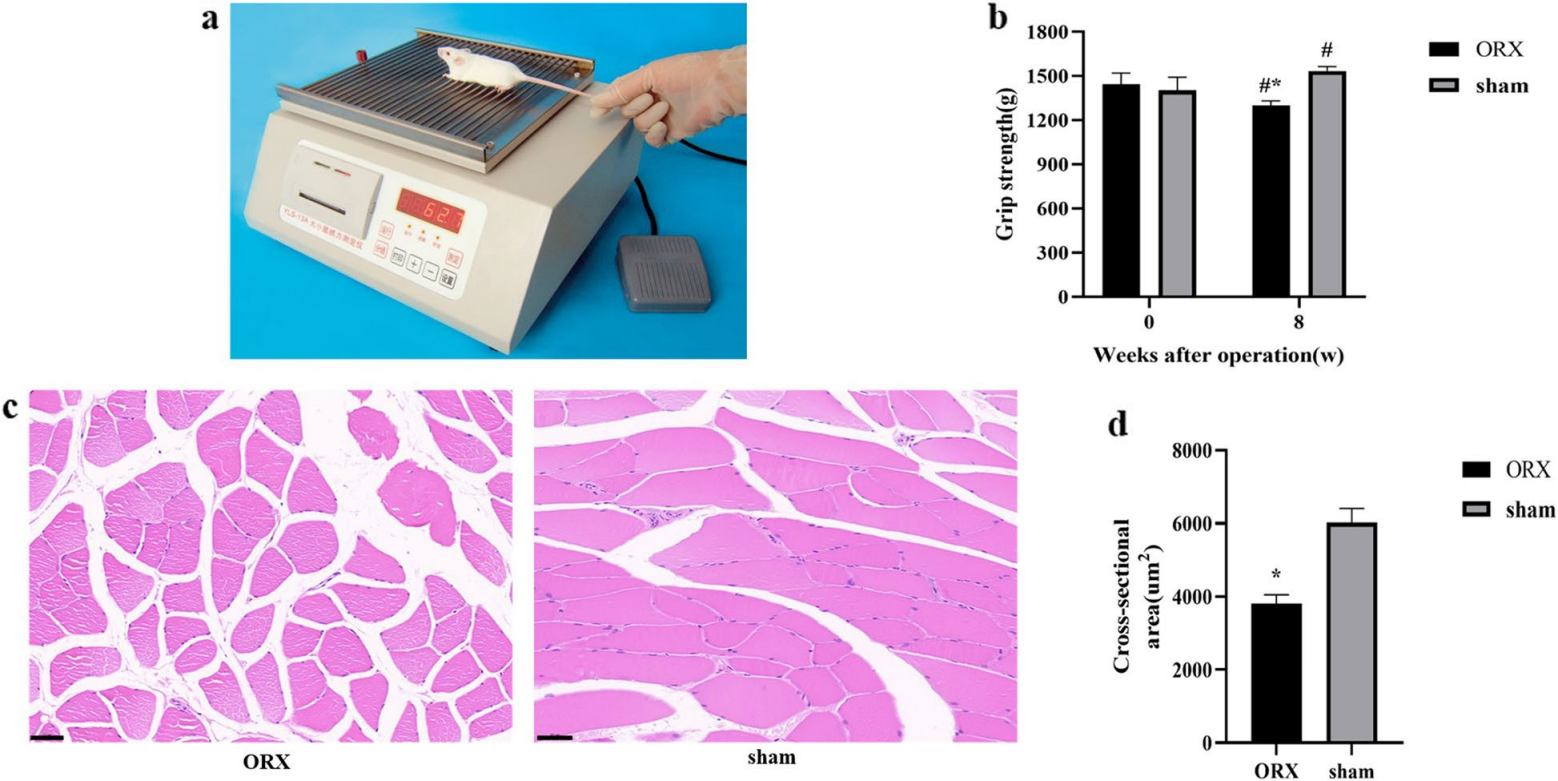
Grip strength and muscle histological results. **a** YLS-13A grip strength tester. **b** Grip strength of ORX and sham rats at baseline and before execution. **c** H&E staining of the soleus muscle in the ORX and sham group. **d** Cross-sectional area of the soleus muscle in the ORX and sham group. *: *P* < 0.05 ORX vs the sham group after 8 weeks of operation. #: *P* < 0.05 vs baseline. Data expressed as mean ± standard error of the mean (SEM). Scale bars indicate 50 µm. Abbreviations: ORX: orchiectomy

At the baseline, the grip strength was 1443.8 ± 75.9 g and 1401.3 ± 90.8 g in ORX and sham rats, without significant difference between the two groups (Fig. 3b). After 8 weeks of ORX or sham operation, the ORX rats had significant weaker grip strength (1298.4 ± 32.5 g) than baseline (*P* < 0.05), while there was a significant higher grip strength (1534.7 ± 29.8 g) in sham rats than baseline (*P* < 0.05). The grip strength of the ORX group was significantly lower than that of sham rats after 8 weeks of operation (Fig. 3b, *P* < 0.05).

The histological investigation declared that the cross-sectional area of the soleus muscle of ORX rats was significantly lower than that of sham rats at execution (3807.6 ± 242.8 um^2^ in ORX rats vs. 6025.4 ± 383.2 um^2^ in sham rats, *P* < 0.05, Fig. 3c).

### Correlations between muscle and bone properties after ORX or sham surgery

**Fig. 4:**
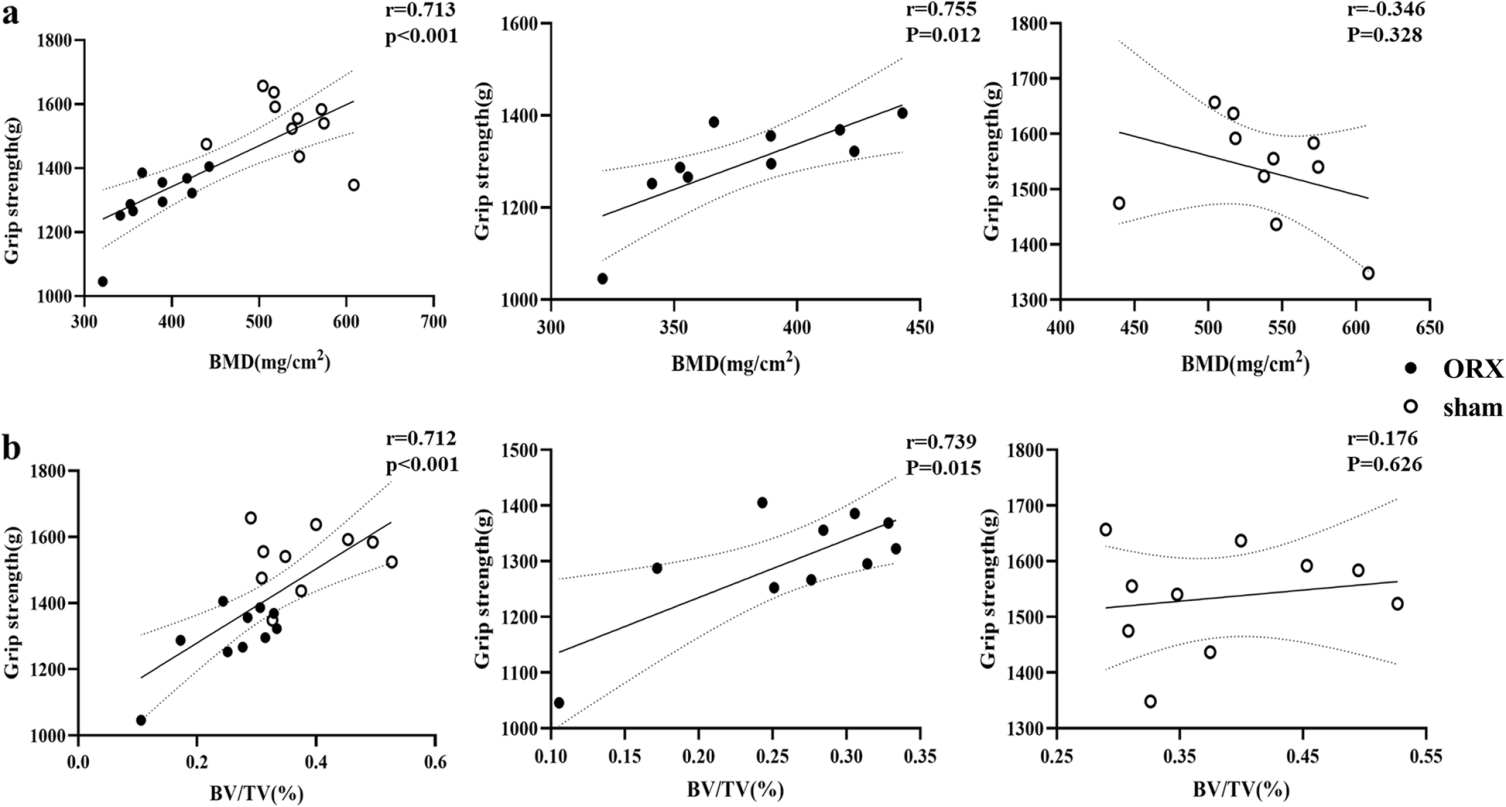
Correlations between sclerostin, irsin and muscle and bone after 8 weeks of ORX or sham operation. **a** Correlation between grip strength and BMD in total rats or ORX or sham rats. **b** Correlation between grip strength and BV/TV in total rats or ORX or sham rats. Abbreviations: BMD: bone mineral density; BV/TV: bone volume per total volume; ORX: orchiectomy

There was a positive correlation between grip strength and femoral trabecular BMD (*r* = 0.713, *P* < 0.001) in all rats with or without ORX (Fig. 4a), which remained significant after adjusting for body weight. When we examined the correlations in ORX group and in sham group separately, a positive correlation between grip strength and trabecular BMD (*r* = 0.755, *P* = 0.012) was found in the ORX rats, while no significant correlations were observed between grip strength and trabecular BMD in sham rats (*r* = -0.346, *P* = 0.328).

In all rats, grip strength and BV/TV showed a significant positive correlation (*r* = 0.712, *P* < 0.001, Fig. 4b), and this correlation remained significant after adjusting for body weight. In ORX group, grip strength positively correlated with BV/TV (*r* = 0.739, *P* = 0.015), but it was not significant in sham group (*r* = 0.176, *P* = 0.626).

### Correlations between serum sclerostin level with properties of muscle and bone after ORX

**Fig. 5:**
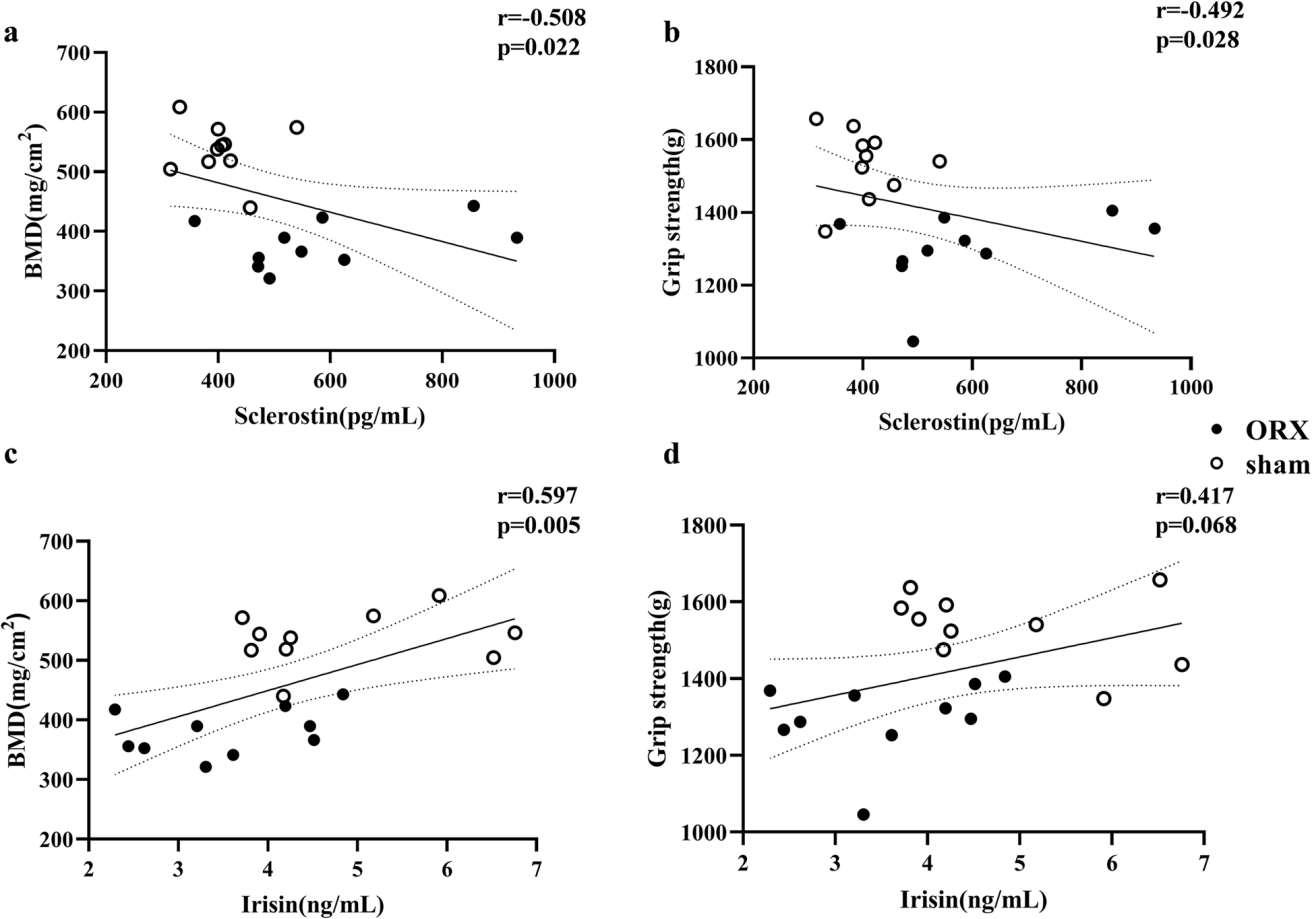
Correlations between serum sclerostin and irisin level with grip strength and BMD aftre 8 weeks of ORX or sham operation. **a** Correlation between serum sclerostin level and BMD total rats. **b** Correlation between serum sclerostin level and grip strength in total rats. **c** Correlation between serum irisin level and BMD in total rats. **d** Correlation between serum irisin level and grip strength in total rats. Abbreviations: BMD: bone mineral density; BV/TV: bone volume per total volume; ORX: orchiectomy

The serum level of sclerostin was negatively correlated with trabecular BMD (*r* = -0.508, *P* = 0.022, Fig. 5a) and muscle strength (*r* = -0.492, *P* = 0.028, Fig. 5b) of total rats after 8 weeks of surgery. However, no statistically significant correlations were found between sclerostin and trabecular BMD neither in ORX rats (*r* = 0.333, *P* = 0.347) nor in sham rats (*r* = -0.090, *P* = 0.805). There was also no obvious correlation between sclerostin and grip strength in ORX (*r* = 0.374, *P* = 0.287) or sham (*r* = -0.031, *P* = 0.931) groups.

### Correlations between serum irisin level with muscle and bone after ORX

In total rats, a positive correlation was observed between the serum level of irisin and trabecular BMD (*r* = 0.597, *P* = 0.005, Fig. 5c), while there was no significant correlation between serum level of irisin and grip strength (*r* = 0.417, *P* = 0.068, Fig. 5d) after 8 weeks of operation. When we analyzed the correlations in each group, no significant correlations between serum irisin and trabecular BMD were observed. Moreover, no significant relationships were found between serum irisin level and grip strength in ORX rats or in sham rats.

## Discussion

In this study, a rat model of androgen deprivation was successfully generated by ORX, and the androgen absence was related to low trabecular BMD, impaired bone microarchitecture, reduced bone strength and grip strength, thin myofibers, which indicated features of osteoporosis and sarcopenia. Serum sclerostin level was significantly increased and irisin level was significantly decreased in ORX rats compared with sham rats after 8 weeks of operation. The grip strength was positively correlated with trabecular BMD and BV/TV in all rats. Sclerostin levels were negatively correlated with trabecular BMD and grip strength of all rats, and serum irisin level was positively correlated with trabecular BMD, but without correlation with grip strength in all rats. No significant correlation of serum sclerostin and irisin levels with BMD, strength of bone and muscle in rats of ORX or sham, and we speculated that this might be related to the small sample size of a single group.

The study measured serum levels of ALP, CTX-I, Ca, P, and Cr and compared their levels between rats of ORX and sham group. Serum levels of ALP and CTX-I were significantly higher in ORX rats than in sham rats, which was similar to the result of a previous study [25]. In this study, ORX led to significant adverse effects on bone volume (BV/TV and Tb.N) and micro-structure (Tb.Sp and Tb.Pf), but not on Tb.Th. The results were consistent with previous studies [26, 27]. And similar results were obtained [28], of which they found that BV/TV, Tb.N and Tb.Sp of ORX rats were significantly affected as early as 4 weeks after ORX, but Tb.Th was unchanged after 16 weeks of ORX. Reduced bone strength and stiffness was observed in ORX rats compared with sham rats, indicating the impaired biomechanical properties. The results were supported by a previous study that the ultimate displacement and stiffness was significantly lower in nine-month-old ORX Wistar rats than sham rats, but no difference of ultimate force was observed between the two groups [29]. This difference might be caused by different age and species of the rats being studied. The Von Kossa’s and H&E staining images displayed the impaired histological characters of femur and vertebra, which were consistent with previous studies in mice [30, 31]. Androgen deficiency by ORX reduced the grip strength and the muscle mass, which was also consistent with the previous study in mice [31].

It was widely accepted that muscle and bone was tightly coupled, and various factors could affect crosstalk between bone and muscle, including mechanical interaction, biochemical coupling, ageing, circadian rhythm, nervous system network, nutrition intake, exosomes and so on. Both bone and skeletal muscle had been identified as secretory endocrine organs, and osteokines and myokines had regulatory effects on a range of tissues through the autocrine, paracrine, and endocrine mechanisms [32, 33]. Sclerostin, an osteogenic factor mainly secreted by mature osteocytes, could inhibit bone formation through binding to the low-density lipoprotein receptor-related protein 4/5/6 Wnt co-receptors in osteoblast [34]. Studies revealed that the loss of sclerostin expression resulted in a high bone mass and increased bone strength in patients with sclerosteosis [35] and Van Buchem disease [36] as well as in sclerostin-deficient mice [37]. The Wnt signaling system was also thought to function in muscles although the underlying mechanism by which sclerostin modulating muscle mass was unclear yet [38]. Wnt‐3a, which was expressed in osteocytes, was found to enhance C2C12 cell differentiation through mechanisms associated with stimulation of the Wnt/β-catenin pathway and up‐regulating the expression of MyoD and Myogenin. Further, a previous study indicated that sclerostin inhibited the effects of Wnt‐3a on the C2C12 myoblast differentiation [14]. Thus, sclerostin might also negatively affect skeletal muscle. A clinical study showed that there was a negative correlation between serum sclerostin levels and muscle mass in non-diabetic men and women in Korea [39]. Several monoclonal antibodies against sclerostin (Scl-Abs) had been developed as potential therapeutic agent for osteoporosis. In a rat model of male osteoporosis, sclerostin antibody significantly increased bone mass and maintained bone quality by increasing bone formation and decreasing bone resorption [40]. Another study revealed that sclerostin inhibition alleviates breast cancer–induced bone metastases and muscle weakness [41], which indicated that sclerostin not only inhibited bone formation, but also might have a negative effect on muscle. In our study, serum sclerostin level was significantly increased in ORX rats compared with sham rats after 8 weeks of operation, which were negatively correlated with trabecular BMD and grip strength of total rats. It was suggested that sclerostin could be an important endocrine regulatory factor and potential therapeutic target of osteoporosis and sarcopenia in men.

Moreover, irisin is a hormone-like myokine secreted abundantly by skeletal muscle in response to physical exercise, both in mice and humans [18]. The previous studies had indicated that irisin could increase bone mass [42, 43]. As for the mechanism, irisin might increase the proliferation, differentiation, and mineralization of osteoblasts through activating the Wnt/β-catenin pathway and p38 mitogen-activated protein kinase and extracellular signal-regulated kinase (p38/ERK MAPK) signaling pathway. Increased expression of RUNX2, which was an important osteogenic marker, and downstream stimulation of ALP and OC were found in the the Wnt/β-catenin and p38/ERK MAPK signaling pathway [44]. Moreover, irisin could reduce osteoclastogenesis through decreasing RANKL-induced osteoclast differentiation and NFAT1c signaling pathway [45]. In addition, treatment of osteocytes with r-irisin could reduce the hydrogen peroxide (H2O2)-induced apoptosis and improve bone remodeling [46]. Furthermore, many researchers examined the relation between irisin level and BMD in humans. A study demonstrated that serum irisin levels had a positive link with femoral and vertebral BMD of older adults, and serum irisin levels was decreased with the onset of bone loss [47]. Thus, irisin might be a potential myokine contributing to regulate bone homeostasis under conditions of bone loss. Moreover, irisin synthesis, by enhancing the expression of the precursors from whose cleavage it derived, might exert its autocrine action in muscle. In vitro, C2C12 myotubes treated with recombinant irisin showed an increased expression of specific mitochondrial transcription factors, which were involved in increasing mitochondrial content and oxygen consumption [48]. In vivo, it was reported that mice treated with recombinant irisin displayed a higher number of fibronectin type III domain containing protein 5 positive fibers than control mice, suggesting that irisin secretion might be amplified by its autocrine action in muscle [42]. In our study, serum irisin level was significantly decreased in ORX rats compared with sham rats after 8 weeks of operation, and a positive correlation was observed between the serum level of irisin and trabecular BMD in total rats of ORX and sham operation, which were consistent with previous studies [49]. The previous study indicated that a decrease in irisin was involved in the muscle/bone relationships in sarcopenia and osteopenia induced by hindlimb unloading and bilateral sciatic neurectomy in mice [49]. Therefore, irisin might be a potential biomarker and a promising therapeutic target for osteoporosis and sarcopenia in men.

Our study investigated in detail the changes of sclerostin and irisin in ORX process of male rats and their relationship with properties of bone and muscle, indicating for the first time that they might be important endocrine factors involved in sarcopenia and osteoporosis in men. However, this study still had several limitations. Firstly, we could not find a causality between sclerostin and irisin level and muscle and bone because we just did simple linear regression. Secondly, we only evaluated the grip strength and muscle histology, but we didn’t evaluate the muscle mass of the rats. Thirdly, we found the correlations between sclerostin, irisin and muscle and bone in total rats. When we did the correlation analysis separately in ORX or sham group, no significant correlations were found between serum levels of sclerostin, irisin and muscle and bone. A possible explanation for this result might be the relatively small sample size in single group. Another limitation of this study was the rats were young and the bones were not yet mature. This might have an impact on results of bone in this study. Lastly, the current mechanism exploration was relatively simple, we didn’t explore the exact changes in expression levels of multiple target genes of Wnt/β‐catenin pathway, RANKL/NFATc1 pathway, p38/ERK MAPK signaling pathway. Further deep-going studies focus on the roles of sclerostin and irisin in the above pathways were needed to be conducted.

In conclusion, we demonstrated that androgen deficiency would impair the microstructure and function of bone and muscle, leading to osteoporosis and sarcopenia. Circulating sclerostin and irisin levels might involve in pathogenesis of osteoporosis and sarcopenia, which could be novel biomarkers and promising therapeutic targets for osteoporosis and sarcopenia in men.

## Supplementary Information

**Additional file 1.**

## Data Availability

All datasets used and/or analyzed during the current study are available from the corresponding author on reasonable request.

## Abbreviations

ORX: Orchiectomy
T: Serum testosterone
Ca: Serum calcium
Cr: Creatinine
P: Serum phosphate
ALP: Alkaline phosphatase
CTX-I: C-telopeptide of type I collagen
BV/TV: Bone volume per total volume
Tb.N: Trabecular number
BS/BV: Bone surface area per bone volume
Tb.Th: Trabecular thickness
Tb.Sp: Trabecular separation
Tb.Pf: Trabecular pattern factor
Ct.Th: Cortical thickness
BMD: Bone mineral density
SEM: Standard error of the mean
H&E: Hematoxylin and eosin
TRAP: Tartrate resistant acid phosphatase
